# EZH2-Inhibited MicroRNA-454-3p Promotes M2 Macrophage Polarization in Glioma

**DOI:** 10.3389/fcell.2020.574940

**Published:** 2020-12-09

**Authors:** Bin Qi, Cheng Yang, Zhanpeng Zhu, Hao Chen

**Affiliations:** Department of Neurosurgery, The First Hospital of Jilin University, Changchun, China

**Keywords:** glioma, EZH2, m^6^A modification of PTEN, YTHDF2, microRNA-454-3p, M2 macrophage polarization

## Abstract

Glioma is a primary intracranial tumor with high incidence and mortality. The oncogenic role of EZH2 has been reported in glioma. EZH2 inhibited microRNA-454-3p (miR-454-3p) by binding to its promoter in chondrosarcoma cells. Therefore, our study aimed to identify whether EZH2 regulated M2 macrophage polarization in glioma via miR-454-3p. Clinical samples of different grades of glioma and glioma cells were collected and immunohistochemistry and RT-qPCR demonstrated that EZH2 was highly expressed in glioma tissues. Expression of EZH2 was positively correlated with the degree of M2 macrophage polarization in glioma tissues. EZH2 was silenced by lentivirus in glioma cells, which were subsequently co-cultured with macrophages to evaluate its effect on macrophage polarization. miR-454-3p, a down-regulated miR in glioma, was found to be increased after silencing of EZH2. Furthermore, MethPrimer analysis showed that EZH2 silencing inhibited the DNA methylation level of miR-454-3p. Additionally, MS-PCR, dual-luciferase reporter, RIP and RNA pull down assays revealed that miR-454-3p promoted PTEN expression by inhibiting m^6^A modification through binding to the enzyme YTHDF2. Either inhibition of miR-454-3p or PTEN resulted in promotion of M2 macrophage polarization. Collectively, histone methyltransferase EZH2 inhibited miR-454-3p through methylation modification and promoted m^6^A modification of PTEN to induce glioma M2 macrophage polarization.

## Introduction

As the most prevalent primary intracranial tumor, glioma accounts for 81% malignant brain tumors (Morgan, 2015). Glioma generally stems from glial progenitor cells and glial cells, and mainly encompasses ependymoma, oligodendroglioma, glioblastoma, astrocytoma, and mixed glioma (Agarwal et al., 2011). Currently, glioma can be treated with maximal safe resection, followed by combined radio-chemotherapy with the alkylating agent Temozolomide (Gusyatiner and Hegi, 2018). As cell components of the natural immune system, macrophages exist in almost all tissues and contribute to immune, repair and homeostasis (Gentek et al., 2014). There is a fact that up to 30–50% of the cells in glioma are microglias or macrophages, which increases the possibility of targeting macrophages as adjuvant therapy for glioma (Hambardzumyan et al., 2016). Importantly, macrophage M1 polarization in combination with immunovirotherapy and immune checkpoint blockade aids to eradication of glioblastoma (Saha et al., 2017). Furthermore, M2 tumor-associated macrophages exert promotive role in the glioma cell growth through releasing cytokines such as interleukin-8 (IL-8), macrophage inflammatory protein-3α (MIP-3α) and interleukin 1β (IL-1β) (Xu Y. et al., 2019; Lu et al., 2020). Therefore, it is indispensable to deep the understanding of molecular mechanism underlying macrophage polarization in glioma.

Enhancer of zeste homolog 2 (EZH2), a histone methyltransferase subunit of polyclonal repression complex, is mutated repeatedly or overexpressed in multiple cancers (Kim and Roberts, 2016). Of note, EZH2 acts as a key mediator of macrophage activation and deletion of EZH2 potentially prevents macrophage-dependent disease development (Neele and de Winther, 2018). Moreover, a research by Ma et al. (2019) has revealed that EZH2 depletion can reduce glioma cell proliferation and tumor formation, yet its effects on the macrophage polarization in glioma remains unknown. Interestingly, a previous study has elucidated that when EZH2 is recruited into the microRNA-454-3p (miR-454-3p) promoter regions, DNA methylation of miR-454-3p is induced to downregulate miR-454-3p in chondrosarcoma cells (Bao et al., 2017). Furthermore, the oncogenic role of miR-454-3p has been reported in glioma development (Shao et al., 2019). Our study predicted the binding relationship between miR-454-3p and YT521-B homology (YTH) domain-containing family protein 2 (YTHDF2) prior to experiments. YTHDF2 expression has been reported to be positively correlated with World Health Organization (WHO) grade of glioma (Chai et al., 2019). As a N6-methyladenosine (m^6^A)-binding protein, YTHDF2 controls the localization and stability of its targeted mRNA (Yu et al., 2019). A recent study suggests that phosphatase and tensin homolog (PTEN) mRNA degradation depends on the m^6^A reader protein YTHDF2 in gastric cancer cells (Yan et al., 2020). Additionally, PTEN downregulation promotes M2 macrophage polarization in the glioma microenvironment and hence facilitates the progression of glioma (Bao and Li, 2019). Considering these findings, a hypothesis was drew that EZH2 might participate in macrophage polarization in glioma via miR-454-3p/YTHDF2/PTEN axis. Hence, experiments were conducted at tissue, cell, and animal levels in this study to verify this hypothesis, thus providing a promising target for glioma treatment.

## Materials and Methods

### Ethics Approval and Consent to Participate

The ethics committee of the First Hospital of Jilin University provided Ethical Approval for the experiments involved human beings in this study which were guided by the intentions of the *Declaration of Helsinki*. Ethical agreements were obtained from the donors or their relatives by written informed consent. The experiments involved animals were implemented in accordance with the principles embodied in the National Institutes of Health Guide for the Care and Use of Laboratory. Efforts were made to avoid all unnecessary distress to the animals.

### Sample Collection

Totally, 90 cases of glioma tissue specimens were collected from the Neurosurgery Department of the First Hospital of Jilin University between January 2014 and January 2017 (the number of cases was determined according to the actual situation). The specimens were pathologically confirmed as primary glioma specimens. According to the WHO (2000) grading criteria for gliomas, there were 30 cases of grade II, III and IV gliomas respectively (the number of cases was determined according to the actual situation). Thirty normal brain tissue specimens obtained by intracranial decompression surgery after brain swelling and herniation following traumatic brain injury were used as normal controls. All specimens were immediately frozen in liquid nitrogen and then transferred to a −80°C refrigerator for storage.

### Reverse Transcription Quantitative Polymerase Chain Reaction (RT-qPCR)

Total RNA was harvested using TRIZOL (15596026, Invitrogen, Carlsbad, CA, United States) and reverse-transcribed into cDNA in the light of the manuals provided by a PrimeScript RT reagent Kit (RR047A, Takara, Tokyo, Japan) and High-Capacity complementary DNA (cDNA) Reverse Transcription Kit (4368813, Thermo Fisher Scientific, Waltham, MA, United States). RT-qPCR was conducted on the obtained cDNA using ABI PRISM 7300 RT-PCR system (Applied biosystems, Foster City, CA, United States) based on the protocols of a Fast SYBR Green PCR kit (Applied biosystems). The relative expression level of mRNA or miR was normalized to glyceraldehyde-3-phosphate dehydrogenase (GAPDH) or U6 expression and was calculated using the 2^–ΔΔCt^ method. The detailed sequences are shown in Table 1.

**TABLE 1:** Primer sequences for reverse transcription quantitative polymerase chain reaction.

**Targets**	**Forward**	**Reverse**
EZH2	5’-AATCAGAGTACATGCGACTGAGA-3’	5’- GCTGTATCCTTCGCTGTTTCC-3’
miR-454-3p	5’-ACCCTATCAATATTGTCTCTGC-3’	5’-GCGAGCACAGAATTAATACGAC-3’
PTEN	5’-CCCACCACAGCTAGAACTTATC-3’	5’-TCGTCCCTTTCCAGCTTTAC-3’
IL-8	5’-CACAAGAGCCAGGAAGAAAC-3’	5’-CTACAACAGACCCACACAATAC-3’
MIP-3α	5’-GCAAGCAACTTTGACTGCTG-3’	5’-CAAGTCCAGTGAGGCACAAA-3’
IL-6	5’-GCTTACCAGGCAACAT-3’	5’-CTGGCACCAGAAACGA-3’
YTHDF2	5’-TAGCCAACTGCGACACATTC-3’	5’-CACGACCTTGACGTTCCTTT-3’
II1b	5’-CTGGTGTGTGACGTTCCCATTA-3’	5’-CCGACAGCACGAGGCTTT-3’
Cd86	5’-TCTCCACGGAAACAGCATCT-3’	5’-CTTACGGAAGCACCCATGAT-3’
Nos2	5’-GTTCTCAGCCCAACAATACAAGA-3’	5’-GTGGACGGGTCGATGTCAC-3’
Cd163	5’-TCCACACGTCCAGAACAGTC-3’	5’-CCTTGGAAACAGAGACAGGC-3’
Ym1	5’-CACCATGGCCAAGCTCATTCTTGT-3’	5’-TATTGGCCTGTCCTTAGCCCAACT-3’
Mrc1	5’-TTGGACGGATAGATGGAGGG-3’	5’-CCAGGCAGTTGAGGAGGTTC-3’
U6	5’-GCGCGTCGTGAAGCGTTC-3’	5’-GTGCAGGGTCCGAGGT-3’
GAPDH	5’-ATGTCGTGG AGTCTACTGGC-3’	5’-TGACCTTGCCCACAGCCTTG-3’

### Cell Treatment and Infection

Normal brain tissue cell HEB, human glioma cell line A172 and human myeloid leukemia mononuclear cell THP-1 (all from Wuhan Bafei Biotechnology Co., Ltd., Wuhan, China) were cultured in Roswell Park Memorial Institute (RPMI)-1640 (Gibco, Carlsbad, CA, United States) containing 10% fetal bovine serum (FBS, Gibco), 10 g/mL streptomycin, and 100 U/mL penicillin, and positioned in a 5% CO_2_ incubator (Thermo Fisher Scientific) at 37°C. The cells in the logarithmic phase were trypsinized, followed by 24-h incubation in 12-well plates at 8 × 10^4^ cells per well. The 75% confluent cells were infected with lentivirus (Lenti) according to the instructions. The silencing sequences targeting EZH2 and YTHDF2, namely, Lenti-EZH2 and Lenti-YTHDF2, were constructed, and the specific silencing sequences are depicted in Table 2. Lenti-HK control with green fluorescent protein (GFP) and resistance to puromycin was purchased from Hanbio Biotechnology (Shanghai, China).

**TABLE 2:** Names and sequences.

**Name**	**Sequences**
MiRZIP-454	5’-ACCCUAUAAGCAAUAUUGCACUA-3’
Lenti-PTEN	5’-CGCGTCCCCGCCAAATTTAACTGCAGAGTTCAAGAGACTCTGCAGTTAAATTGGCTTTTTGGAAAT-3’
Lenti-YTHDF2	5’-AAGGACGTTCCCAATAGCCAA-3’
Lenti-EZH2	5’-CCGGCCCAACATAGATGGACCAAATCTCGAGATTTGGTCCA TCTATGTTGGGTTTTTG-3’
Lenti-HK	5’-TTCTCCGAACGTGTCACGTTT-3’

### Flow Cytometry

Following overnight culture in 60 mm cell culture dish at 4 × 10^5^ cells/well, cells were treated with corresponding drugs. The cells were washed with 1 mL phosphate buffer saline (PBS) and transferred into a 15-mL tube. The supernatant was discarded following 5-min cell centrifugation at 800 rpm. Cells were re-suspended in 5 mL PBS, and centrifuged again, followed by removal of supernatant. This procedure repeated two times. The cells were then resuspended in 0.1 mL PBS and transferred to a 1.5-mL centrifuge tube. The 1 μL primary antibody was supplemented into cells at ratio of 1: 100 for 1–2 h of incubation. Cells were centrifuged at 1,500 rpm for 5 min, followed by discarding of the supernatant. After re-suspension in 1 mL PBS, cells were centrifuged, and the supernatant was discarded, which was repeated three times. The washed cells were re-suspended in 0.1 mL PBS. The cells suspension was added with flow antibodies (Proteintech Group Inc., Wuhan, China) to CD206 (1: 1,000, 18704-1-AP) and CD11b (1: 1,000, APC-65116) for 1-h incubation at room temperature. The supernatant was removed after 5-min cell centrifugation at 15,000 rpm. After addition of 1 mL PBS, cells were re-suspended, and centrifuged with supernatant discarded, which was repeated three times. The cells were resuspended in 0.2 mL PBS, wrapped in tin foil to avoid light, and added to flow tubes for analysis on an EPICS_XL flow cytometer.

### Enzyme-Linked Immunosorbent Assay (ELISA)

Tissue preserved in a -80°C freezer was obtained, and homogenized using normal saline to prepare 10% tissue homogenate (1 μg brain tissues were dissolved in 9 μL). After sample centrifugation at 14,000 g, the supernatant was harvested. On the basis of instructions, cytokine production in tumor tissues and supernatants of culture media was detected using the IL-8 (E-EL-H0048c), MIP-3α (E-EL-H0027c), and IL-6 (E-EL-H0102c) ELISA kits from Elabscience Biotechnology Co., Ltd. (Wuhan, China).

### Dual-Luciferase Reporter Assay

The 2,000 base sequences (i.e., promoter region) in upstream of PTEN or YTHDF2 transcription start point were cloned into pGL3.0-basic vector (OBiO Technology, Shanghai, China) to construct fluorescent plasmids. One portion of fluorescent plasmids was co-treated into A172 cells with the miR-454-3p overexpression plasmid or EZH2 overexpression plasmid, and the other portion was co-treated into A172 cells with the miRZIP-454 overexpression plasmid or NC plasmid. After 48-h treatment, the luciferase activity was measured by Promega system (Promega, Madison, WI, United States).

The YTHDF2 3′-untranslated region (3′-UTR) fragment containing the putative binding site of miR-454-3p was amplified by PCR and inserted into the p-MIR-reporter plasmid, namely YTHDF2 wild type (WT). After site-directed mutagenesis, the fragment was inserted into the p-MIRreporter plasmid to construct YTHDF2 mutant type (MUT). Plasmids were verified by DNA sequencing. THP1 cells were cultured in a 24-well plate (5 × 10^4^ cells/well), and then each well was co-treated with YTHDF2 WT or YTHDF2 MUT and miR-454-3p, miRZIP-454 or NC and β-galactosidase (β-gal) expression plasmids using Lipofectamine^TM^2000. The β-gal expression plasmid was detected as a treatment control. Cells were collected 48 h after treatment and detected using a luciferase detection kit.

### Hematoxylin-Eosin (HE) Staining

Tissue blocks preserved in 4% paraformaldehyde were collected, embedded in paraffin, and cut into 4 μm slices for histological analysis by HE staining. Cross-sectional images were obtained by Leica Microsystems (DM2000, CMS GmbH, Wetzlar, Germany). Six fields of view were randomly selected from each section to evaluate the establishment of a glioma model in nude mice.

### Immunohistochemistry

Tissue blocks were prepared into 4 μm paraffin-embedded slices. The primary antibodies (Proteintech Group Inc.) to EZH2 (1: 500), CD206 (1: 500), PTEN (1: 500, 22034-1-AP), and YTHDF2 (1: 500, 24744-1-AP) were supplemented for slice incubation, followed by slice culture with horseradish peroxidase (HRP)-tagged secondary antibodies. Diaminobenzidine staining was performed. The slices were counterstained with hematoxylin. Cross-sectional images were obtained by Leica Microsystems (DM2000, CMS GmbH, Wetzlar, Germany). Six fields of view per section were randomly selected by ImagePro Plus 6.0 (Media Cybernetics, Inc., Rockville, MD, United States) for immunohistochemical scoring.

### Immunofluorescence

After washing and sealing, the paraffin-embedded slices (4 μm) were incubated with primary antibodies (Proteintech Group Inc.) to CD206 (1: 500, 18704-1-AP), IL-8 (1: 500, 27095-1-AP), MIP-3α (1: 500, 26527-1-AP), and IL-6 (1: 500, 21865-1-AP), followed by incubation with Cy3-conjugated goat anti-rabbit Immunoglobulin G (IgG) or AlexaFluor #488-conjugated AffiniPure goat anti-mouse IgG secondary antibodies. The samples were immersed in sealant containing 4′-6-diamidino-2-phenylindole (DAPI). Sections were scanned by a digital slide scanner (Pannoramic MIDI, 3DHISTECH Co., Ltd., Budapest, Hungary). The images were analyzed by 3D HISTECH Pannoramic viewer.

### Western Blot Analysis

Cells were harvested by trypsin treatment and lysed with enhanced radio-immunoprecipitation assay (RIPA) lysis buffer (BOSTER Biological Technology Co., Ltd., Wuhan, Hubei, China) containing protease inhibitors. A bicinchoninic acid protein assay kit (BOSTER Biological Technology Co., Ltd.) was employed for estimation of protein concentration. The protein underwent separation by 10% sodium dodecyl sulfate polyacrylamide gel electrophoresis, and electroblotted to a polyvinylidene fluoride membrane which received 2-h sealing by 5% bovine serum albumin at room temperature to block nonspecific binding. After supplement with primary antibodies (Proteintech Group Inc.) to EZH2 (1: 2,000, 21800-1-AP), PTEN (1: 2,000, 22034-1-AP), YTHDF2 (1: 1,000, 24744-1-AP), and GAPDH (1: 5,000, 60004-1-Ig) and primary antibodies from Abcam (Cambridge, United Kingdom) to N-cadherin (ab76011, 1: 5,000), and Vimentin (ab92547, 1: 2,000), the membrane was incubated at 4°C overnight. Afterward, HRP-tagged goat anti-rabbit secondary antibody (ab205719; 1: 2,000; Abcam, Cambridge, United Kingdom) was supplemented to incubate the membrane at room temperature for 1 h, followed by 1-min membrane incubation with electrogenerated chemiluminescence (ECL) working solution (EMD Millipore, Billerica, MA, United States) at room temperature. The bands of Western blot analysis images were quantified by Image J analysis software, and GAPDH was adopted as the internal reference.

### Tumor Xenograft Mouse Model

Sixteen BALB/c nude mice [Beijing Institute of Pharmacology, Chinese Academy of Medical Sciences (Beijing, China); 4 weeks old] were separately caged in specific pathogen-free animal laboratory. After 1-week acclimation, the experiment was started. The health status of nude mice was observed before the experiment. During the feeding process, nude mice were randomly divided into two groups. Luciferase-labeled A172 cells (1,000,000 cells per mouse) were mixed with polarized macrophages (200,000 cells per mouse), and the mixture was injected into the axilla of nude mice. In detail, two groups of nude mice were injected with pLenti-EZH2-GFP-treated A172 cells and polarized macrophages (*n* = 8) or pLenti-HK-GFP-treated A172 cells and polarized macrophages. Tumor size and body weight of nude mice were measured twice a week, 4 weeks in total. The tumor volume was calculated according to the following formula: (length × width^2^)/2. After 2 nmol tracer was injected into the tail vein, the single Cy5 images of the same site were collected 24 h post-injection and the values were measured with a circular region of interest (ROI) in each image based on the *in vivo* imaging system (IVIS spectroscopy). The luminous intensity was obtained to evaluate the tumor growth. The mice were euthanized after 4 weeks, and the tumors in vivo were obtained for subsequent detection.

### Co-culture of THP-1-Differentiated Macrophages With A172 Cells

THP-1 cells were stimulated with Phorbol ester-12-myristate-13-acetate (PMA, 100 ng/mL, Sigma, St Louis, MO, United States) to induce differentiation into macrophages. Afterward, normal or Lenti-EZH2-treated A172 cells were added to the top of the stratified co-culture Transwell (Corning Incorporated., Corning, N.Y., United States) and co-cultured with macrophages (ratio 2: 1) in 2 mL of 10% Dulbecco’s Modified Eagle Medium for 72 h. Transwell chamber (Transwell insert) was shaped as a small cup that can be placed in the plates, and a permeable membrane was at the bottom of the cup with micropores of size of 0.1–12.0 microns (different materials were used according to different needs). During co-culture, the macrophages at the bottom were unable to contact the A172 cells at the top.

### Methylation Specific PCR (MS-PCR)

MS-PCR was performed to assess the methylation status of PTEN promoter. Initially, glioma DNA was extracted using BGenomic DNA Extraction Kit (Beijing Tiangen Biotech, Beijing, China), DNA concentration and purity were determined with ultraviolet spectrophotometry and stored in −80°C refrigerator for later use. MS-PCR was then conducted with the DNA Methylation-Gold^TM^ kit (D5005, Zymo Research, Irvine, CA, United States). Briefly, 1 μg of DNA was subjected to bisulfite modification, and stored at −80°C for no longer than 1 month, followed by desulfurization and purification using reaction column. The purified DNA can be used for subsequent PCR reaction. PCR reaction conditions were as follows: pre-denaturation at 95°C for 10 min; 35 cycles of denaturation at 95°C for 45 s, methylation at 58°C/non-methylation at 57°C for 45 s, and annealing at 72°C for 45 s; finally extension at 72°C for 10 min. The reaction product was subjected to agarose gel electrophoresis, gel electrophoresis imaging and image analysis. If the CpG island in the PTEN promoter region is completely methylated, only the methylated primer could amplify the target band; if it is completely unmethylated, only the unmethylated primer can amplify the target band; if it is partially methylated, both pairs of primers can amplify the target band. Partial methylation is classified as methylation. Each experiment was repeated three times.

### Promoter CpG Island Prediction of miRNAs

The Genomes function of the database UCSC Genome Atlas^1^ was used to obtain the promoter sequence of human miR-454. Through the online tool MethPrimer^2^, the promoter CpG islands of miR-454 were predicted by selecting Pick primers for bisulfite sequencing PCR or restriction PCR.

### RNA Immunoprecipitation (RIP) and Methylated RNA Immunoprecipitation (MeRIP) Assays

RIP kit (Millipore, United States) and MeRIP kit (Merck Millipore, United States) were adopted to detect the binding of relevant RNA to protein. Cells were lysed in an ice bath with equal volume of RIPA lysis (P0013B, Beyotime Biotechnology Co., Ltd., Shanghai, China) for 5 min, and centrifuged at 14,000 rpm for 10 min at 4°C to obtain the supernatant. One part of the cell extract was utilized as input, and the other part was incubated with antibody for co-precipitation. Samples and Input were digested by proteinase K to extract RNA for subsequent RT-qPCR detection of target RNA. The antibodies (Proteintech Group Inc.) used for RIP and MeRIP were as follows: YTHDF2 (1: 1,000, 15073-1-AP), m^6^A antibody (pAb) (1: 1,000, 61495), and IgG (1: 100, 10285-1-AP). Antibodies were mixed with cells for 30 min at room temperature. Rabbit anti-human IgG served as NC.

### RNA Pull-Down

RNA pull-down experiment was conducted according to manuals of a Pierce^TM^ magnetic RNA pull-down kit (20,164, Thermo Fisher Scientific, Waltham, MA, United States). According to protocols of Ribo^TM^RNAmax-T7 Transcription Kit (RiboBio, Guangzhou, China), the miR-454-3p RNA was transcribed *in vitro* using a DNA template containing the T7 promoter. Then, miR-454-3p was end-labeled with desulfurizing biotin using Pierce RNA 3’end desulfurization biotinylation kit (20,163, Thermo Fisher Scientific). A172 cells were treated with the miR-454-3p plasmid for 48 h and then harvested. Biotinylated miR-454-3p was captured with streptavidin magnetic beads and then mixed with A172 cell extract. The protein was eluted from the RNA-protein complex and detected by western blot analysis using YTHDF2 antibody.

### Chromatin Immunoprecipitation (ChIP)

As per the instructions provided by ChIP kit (Millipore), upon 70–80% confluence, cells were fixed with 1% formaldehyde at room temperature for 10 min to crosslink the DNA and protein in the cells, and added with glycine to the final concentration at 0.125 M to stand at room temperature for 5 min. After cross-linking, the cells were broken into fragments by ultrasonication at 120 w each time, 2 s on, 5 s off, 15 cycles in total, followed by centrifugation at 13,000 rpm at 4°C. The supernatant was collected and divided into three portions, which were incubated with positive control antibody RNA polymerase II, NC antibody normal human IgG (1:100, 10285-1-AP) and rabbit anti-EZH2 (ab191250, 1:100) overnight at 4°C, respectively. The endogenous DNA-protein complex was precipitated with Protein Agarose/Sepharos and centrifuged. The supernatant was aspirated and removed. The non-specific complex was washed, followed by de-crosslinking overnight at 65°C. The DNA fragment was harvested by phenol/chloroform extraction and purification. The expression of the PTEN promoter was tested by RT-qPCR. Each experiment was repeated three times.

### Statistical Analysis

All measurement data were shown as mean ± standard deviation and analyzed by SPSS 21.0 software (IBM, Armonk, NY, United States), with *p* < 0.05 as a level of statistically significance. Data between two groups were compared by unpaired *t*-test, while those among multiple groups were tested using one-way analysis of variance (ANOVA), followed by Tukey’s *post-hoc* test. Comparison of data at different time points was analyzed using two-way ANOVA. Pearson correlation analysis was performed to observe the correlation of indicators.

## Results

### EZH2 Overexpression Was Related to M2 Macrophage Polarization in Patients With Glioma

**FIGURE 1:**
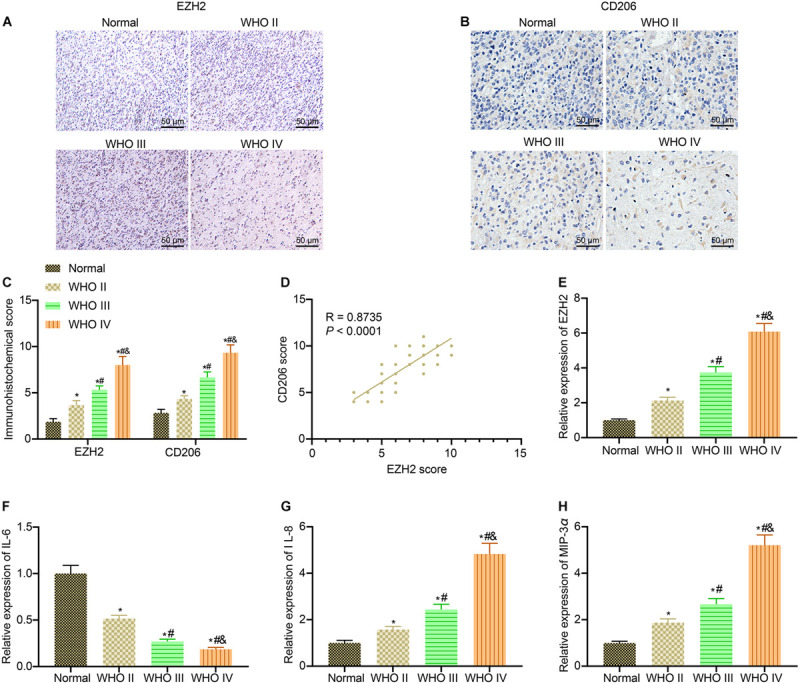
M2 macrophage polarization in patients with glioma is associated with EZH2 overexpression. **(A)** Immunohistochemistry analysis of EZH2 in clinical samples of different grades of gliomas (×200). **(B)** Immunohistochemistry analysis of CD206 in clinical samples of different grades of gliomas (×200). **(C)** EZH2 and CD206 immunohistochemical scores of clinical specimens of different grades of gliomas. **(D)** Pearson correlation analysis of EZH2 and CD206 immunohistochemical scores in glioma clinical samples. **(E)** EZH2 expression in glioma clinical specimens determined by RT-qPCR. **(F)** The expression of IL-6 in glioma clinical specimens determined by RT-qPCR. **(G)** The expression of IL-8 in glioma clinical specimens determined by RT-qPCR. **(H)** The expression of MIP-3α in glioma clinical specimens determined by RT-qPCR. *n* = 30 in WHO II group; *n* = 30 in WHO III group; *n* = 30 in WHO IV group; *n* = 30 in normal group. *p < 0.05 vs. normal brain tissues. ^#^*p* < 0.05 vs. WHO II glioma tissues. ^&^*p* < 0.05 vs. WHO III glioma tissues. All measurement data were shown as mean ± standard deviation. Data between two groups were compared by unpaired *t*-test, while comparisons among multiple groups were performed using one-way ANOVA, followed by Tukey’s *post-hoc* test. Pearson correlation analysis was performed to observe the correlation of indicators.

EZH2 plays a critical part in the development of glioma (Han et al., 2020). Additionally, it was reported that macrophages are involved in glioma development (Pang et al., 2018). Hence, we speculated that EZH2 was associated with macrophage polarization in glioma. Initially, the expression of CD206, a specific marker of M2 macrophages, and EZH2 level in different grades of clinical glioma samples were detected by immunohistochemistry. The results showed that compared with normal brain tissues, the expression of EZH2 and CD206 in glioma clinical samples showed an increasing trend with the elevation of glioma grade (Figures 1A,B). The results of immunohistochemical score displayed that the scores of EZH2 and CD206 were enhanced with the increase of glioma grade in comparison with normal brain tissues (*p* < 0.05; Figure 1C). As expected, through Pearson correlation analysis, we found that the immunohistochemical scores of EZH2 and CD206 showed a positive correlation (Figure 1D). From RT-qPCR results, with the increase of glioma grade, the expression of IL-6 in glioma clinical samples showed a downward trend, while expression of EZH2, IL-8, and MIP-3α exhibited an upward trend (Figures 1E–H). These results suggested that EZH2 upregulation was correlated to M2 macrophage polarization in patients with glioma.

### EZH2 Silencing Inhibits the Growth of Glioma and M2 Macrophage Polarization in Nude Mice

**FIGURE 2:**
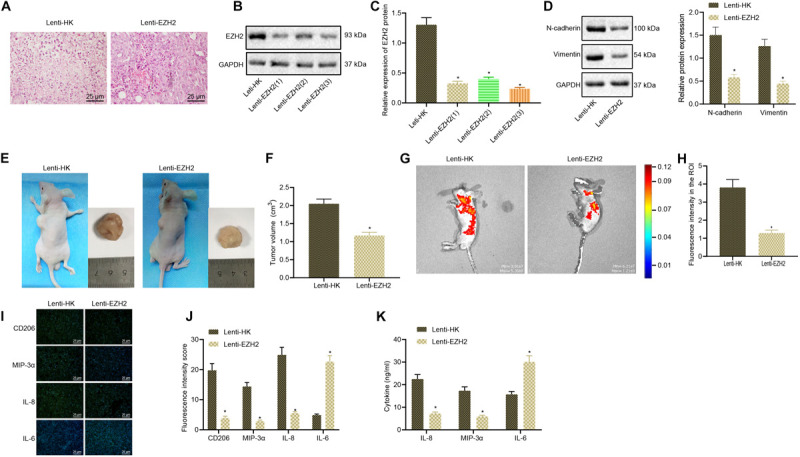
The growth of glioma and M2 macrophage polarization is repressed by silencing EZH2 in nude mice. **(A)** The successful establishment of glioma xenograft model in nude mice determined using HE staining (×200). **(B)** Western blots of EZH2 protein. **(C)** Western blot analysis to verify the silencing efficiency of Lenti-EZH2. **(D)** The expression of N-cadherin and Vimentin proteins measured by Western blots. **(E)** A172 cells treated with Lenti-EZH2 or Lenti-HK were co-injected with polarized macrophages into the axilla of nude mice, and the size of glioma was measured and analyzed 2 months later. **(F)** Tumor size of mice. **(G)** The GFP fluorescence intensity of glioma formation in nude mice measured by an *in vivo* imaging system (IVIS spectrum), and the signal intensity of ROI reflected the growth of the tumor. **(H)** Statistical analysis of ROI signal intensity. **(I)** Immunofluorescence detection of the expression of CD206, MIP-3α, IL-6, and IL-8 in tumor tissues of mice (×400). **(J)** Statistical analysis of immunofluorescence intensity of CD206, MIP-3α, IL-6, and IL-8 in mice. **(K)** The expression of MIP-3α, IL-6, and IL-8 in plasma of nude mice evaluated by ELISA. *p < 0.05 vs. nude mice injected with Lenti-HK-treated glioma cells and polarized macrophages. *n* = 8. All measurement data were shown as mean ± standard deviation. Data between two groups were compared by unpaired *t*-test, while comparisons among multiple groups were performed using one-way ANOVA, followed by Tukey’s *post-hoc* test.

Next, we continued to validate whether EZH2 could promote glioma development by promoting M2 macrophage polarization *in vivo* through tumorigenesis model in nude mice. Glioma cells and macrophages were injected into the axilla of nude mice to induce the *in vivo* xenograft nude mouse model. After 4 weeks of modeling, the collected tumors were stained with HE to verify the success establishment of *in vivo* glioma tumorigenesis model in nude mice (Figure 2A). EZH2 was silenced by lentivirus treatment in glioma cell line A172. Western blot analysis validated the successful silencing of EZH2 and Lenti-EZH2(3) with the best silencing efficiency was selected in the subsequent experiments (Figures 2B,C). EZH2 knocked resulted in reduced expression of N-cadherin and Vimentin (Figure 2D). Both physical and fluorescence imaging results exhibited that silencing EZH2 by lentivirus significantly inhibited tumor size in nude mice (Figures 2E–H). Immunofluorescence results depicted that silencing EZH2 by lentivirus obviously reduced the expression of M2 macrophage polarization-related indicators (CD206, MIP-3α, and IL-8) but potently increased the expression of IL-6 in the glioma tissues of nude mice (Figures 2I,J). The plasma concentrations of MIP-3α, IL-6, and IL-8 were measured by ELISA kit. The results described that EZH2 silencing significantly reduced the secretion of MIP-3α and IL-8 and elevated the secretion of IL-6 in nude mice with glioma (Figure 2K). Collectively, EZH2 silencing decreased the growth of glioma and delayed M2 macrophage polarization in nude mice.

### EZH2 Downregulation Affects the Microenvironment of Glioma and Represses the Polarization of M2 Macrophages

**FIGURE 3:**
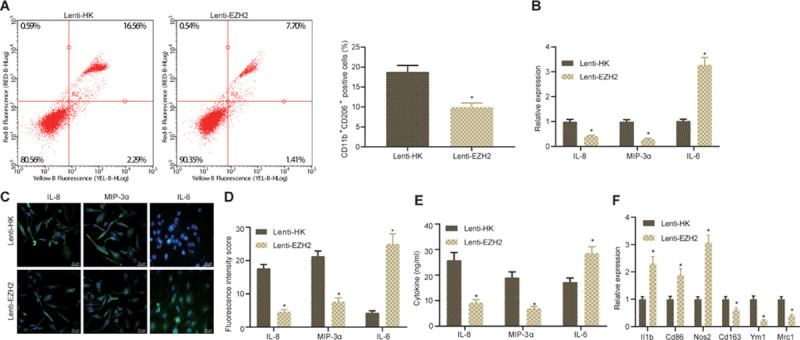
Downregulated EZH2 alters the microenvironment of glioma and suppresses the polarization of M2 macrophages co-cultured *in vitro*. **(A)** The proportion of CD11b^+^ CD206^+^ cells in the co-cultured cells determined by flow cytometry. **(B)** The expression of IL-8, MIP-3α, and IL-6 in macrophages measured by RT-qPCR. **(C)** The expression of IL-8, IL-6, and MIP-3α in macrophages detected by immunofluorescence (×400). **(D)** Statistical analysis of the fluorescence intensity of IL-8, IL-6, and MIP-3α in macrophages. **(E)** The content of IL-8, MIP-3α, and IL-6 in cell culture supernatant detected by ELISA. **(F)** The expression of M1 polarization markers II1b, Cd86, and Nos2 as well as M2 markers Cd163, Ym1, and Mrc1 in macrophages determined by RT-qPCR. *p < 0.05 vs. treatment with Lenti-HK. All measurement data were shown as mean ± standard deviation. Data between two groups were compared by unpaired *t*-test. Experiments were repeated three times independently.

Next, glioma cells were co-cultured with macrophages *in vitro* to study whether the expression of EZH2 in glioma could polarize macrophages into M2. The CD11b^+^ CD206^+^ cells were detected by flow cytometry to determine the proportion of M2 macrophages. As displayed in Figure 3A, infection with Lenti-EZH2 in glioma cells significantly decreased the proportion of co-cultured M2 macrophages. Then, RNA from co-cultured macrophages was extracted, and the expression of MIP-3α, IL-6, and IL-8 in macrophages was detected by RT-qPCR. The results demonstrated that infection with Lenti-EZH2 in glioma cells appreciably reduced the expression of MIP-3α and IL-8 and increased the expression of IL-6 in co-cultured M2 macrophages (Figure 3B). Meanwhile, immunofluorescence depicted that the expression of MIP-3α and IL-8 was strikingly declined but IL-6 expression was remarkably elevated in co-cultured M2 macrophages after infection with Lenti-EZH2 in glioma cells (Figures 3C,D). Then, based on ELISA results, the expression of MIP-3α and IL-8 was prominently diminished but IL-6 expression was severely enhanced in co-culture medium in the presence of Lenti-EZH2 (Figure 3E). Moreover, II1b, Cd86, and Nos2 is reported to serve as M1 polarization markers and Cd163, Ym1, and Mrc1 as M2 markers (Eisel et al., 2019). RT-qPCR was conducted to determine the expression of M1 markers II1b, Cd86, and Nos2 as well as M2 markers Cd163, Ym1, and Mrc1, displaying that infection with Lenti-EZH2 in glioma cells induced potent rise in the expression of II1b, Cd86, and Nos2, and marked reduction in the expression of Cd163, Ym1, and Mrc1 in macrophages (Figure 3F). Collectively, downregulation of EZH2 in glioma cells suppressed the polarization of M2 macrophages.

### EZH2 Enhances Polarization of M2 Macrophages by Inhibiting miR-454-3p Through DNA Methylation

**FIGURE 4:**
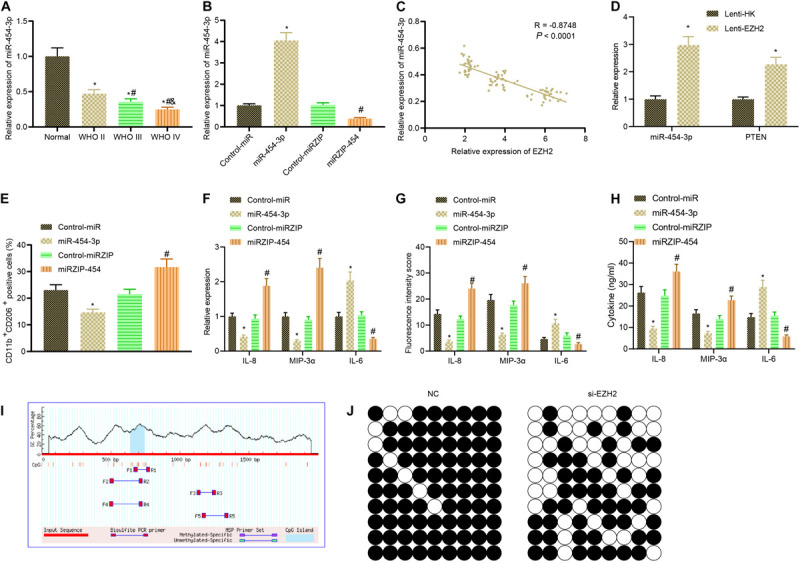
EZH2 contributes to miR-454-3p downregulation via DNA methylation to promote polarization of M2 macrophages. **(A)** The expression of miR-454-3p in clinical samples of different grades of glioma detected by RT-PCR. *p < 0.05 vs. normal brain tissues, ^#^*p* < 0.05 vs. WHO II glioma tissues, ^&^*p* < 0.05 vs. WHO III glioma tissues. **(B)** The efficiency of miR-454-3p and miRZIP-454 verified by RT-qPCR. *p < 0.05 vs. the treatment with control-miR, ^#^*p* < 0.05 vs. the treatment with control-miRZIP. **(C)** Pearson correlation analysis of EZH2 and miR-454-3p expression in glioma clinical samples. **(D)** RT-qPCR to detect the expression of miR-454-3p and PTEN in glioma cells after EZH2 inhibition. *p < 0.05 vs. the treatment with Lenti-HK. **(E)** The proportion of CD11b^+^ CD206^+^ cells in cells after co-culture of miR-454-3p-overexpressed/knockout A172 cells with THP-1 cells detected by flow cytometry. **(F)** The expression of IL-8, IL-6, and MIP-3α in the macrophages after co-culture with A172 cells treated with Lenti-PTEN determined by RT-qPCR. **(G)** Statistical analysis of the fluorescence intensity of IL-8, IL-6, and MIP-3α in the tumor microenvironment. **(H)** The levels of IL-8, IL-6, and MIP-3α in the supernatant detected by ELISA. *p* < 0.05 vs. the treatment with control-miR, ^#^*p* < 0.05 vs. the treatment with control-miRZIP. **(I)** CpG island and primer prediction. **(J)** MethPrime analysis of promoter CpG islands of miR-454-3p. All measurement data were shown as mean ± standard deviation. Data between two groups were compared by unpaired *t*-test, while comparisons among multiple groups were performed using one-way ANOVA, followed by Tukey’s *post-hoc* test. Pearson correlation analysis was performed to observe the correlation of indicators. Experiments were repeated three times independently.

Subsequently, we examined upstream mechanism of EZH2. RT-qPCR illustrated that compared with normal brain tissues, the expression of miR-454-3p in glioma clinical samples showed a downward trend with the increase of glioma grade (Figure 4A). Then, the antisense strand of miR-454-3p, miRZIP-454, was constructed, and it was verified by RT-qPCR that transfection of miR-454-3p into cells significantly increased miR-454-3p expression, while infection of miRZIP-454 reduced the expression of miR-454-3p (Figure 4B). Based on Pearson correlation analysis, a negative correlation was observed between the expression of EZH2 and miR-454-3p (Figure 4C). According to RT-qPCR, after inhibiting EZH2, the expression of miR-454-3p and PTEN was enhanced markedly (Figure 4D). Then, A172 cells were treated with miR-454-3p and miRZIP-454 to construct miR-454-3p-overexpressed/knockout A172 cells which were then co-cultured with THP-1 cells. Flow cytometry documented that overexpression of miR-454-3p noticeably declined but inhibition of miRZIP-454 observably increased the proportion of co-cultured M2 macrophages (Figure 4E). The expression of IL-8, IL-6, and MIP-3α in the macrophages was determined by RT-qPCR, that of IL-8, IL-6, and MIP-3α in the tumor microenvironment detected by immunofluorescence and their levels in the culture medium of co-cultured cells measured using ELISA. Results demonstrated reduced expression of MIP-3α and IL-8 in macrophages, fluorescence intensity, and their levels in the co-culture medium, but increased IL-6 expression in macrophages, fluorescence intensity and its level in the co-culture medium in the presence of miR-454-3p; miR-454-3p silencing resulted in elevated expression of MIP-3α and IL-8 in macrophages, fluorescence intensity, and their levels in the co-culture medium but reduced IL-6 expression in macrophages, fluorescence intensity and its level in the co-culture medium (Figures 4F–H).

It was reported that EZH2 bound to the miR promoter and recruited DNA (cytosine-5-)-methyltransferase 1 (DNMT1) (Ning et al., 2015), which can mediate DNA methylation on CpG islands of its target genes (Fang et al., 2015). DNA methylation levels on CpG islands in the promoter region of miR-454-3p were analyzed using MethPrimer, followed by analysis of methylation level of miR-454-3p DNA after EZH2 inhibition. The results manifested that the methylation levels of miR-454-3p were reduced substantially after EZH2 inhibition by lentivirus (Figures 4I,J). In conclusion, EZH2 induced DNA methylation to downregulate miR-454-3p, thus promoting polarization of M2 macrophages.

### miR-454-3p Elevates PTEN Expression in Glioma Cells

By querying the sequence and potential target genes of miR-454-3p, YTHDF2 was found to be a putative target of miR-454-3p. As an m^6^A reader protein, YTHDF2 recognizes m^6^A and recruits the mRNA decay machinery to increase mRNA degradation (Li et al., 2018). As previously reported, YTHDF2 decreased PTEN expression by recognizing the PTEN m^6^A modification pathway in gastric cancer cells (Yan et al., 2020). Thus, we next explore the correlation among miR-454-3p, YTHDF2, and PTEN in glioma.

**FIGURE 5:**
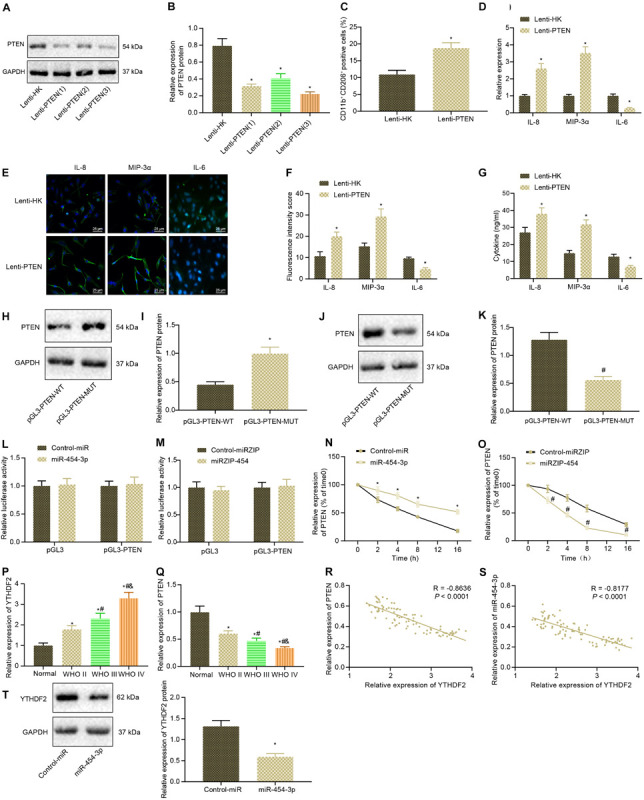
PTEN expression is enhanced by miR-454-3p overexpression in glioma cells. **(A)** The efficiency of Lenti-PTEN determined using western blot analysis. **(B)** The protein expression of PTEN after PTEN knockdown. *p < 0.05 vs. treatment with Lenti-HK. **(C)** The proportion of CD11b^+^ CD206^+^ cells in the co-cultured cells detected by flow cytometry. **(D)** The expression of IL-8, IL-6, and MIP-3α in the macrophages after co-culture with A172 cells treated with Lenti-PTEN determined with RT-qPCR. **(E)** The expression of IL-8, IL-6, and MIP-3α in the tumor microenvironment detected using immunofluorescence. **(F)** Statistical analysis of the fluorescence intensity of IL-8, IL-6, and MIP-3α in the tumor microenvironment. **(G)** The levels of IL-8, IL-6, and MIP-3α in supernatant detected by ELISA. **(H)** Western blots of PTEN after treatment of miR-454-3p into glioma cell. **(I)** Western blot analysis of the protein expression changes of PTEN after treatment of miR-454-3p into glioma cells. **(J)** Western blots of PTEN after treatment of miRZIP-454. **(K)** Western blot analysis of the protein expression changes of PTEN after treatment of miRZIP-454 into glioma cells. *p < 0.05 vs. treatment with control-miR, ^#^*p* < 0.05 vs. treatment with control-miRZIP. **(L)** The binding of miR-454-3p to PTEN after treatment of miRZIP-454 into glioma cells evaluated using a dual luciferase reporter system. **(M)** The binding of miR-454-3p to PTEN after treatment of miRZIP-454 into glioma cells evaluated using a dual luciferase reporter system. **(N)** The expression of PTEN mRNA every 4 h after treatment of miR-454-3p and addition of RNA synthesis inhibitor a-amanitin (50 mM) into glioma cells. **(O)** The expression of PTEN mRNA every 4 h after treatment of miRZIP-454 and addition of RNA synthesis inhibitor a-amanitin (50 mM) into glioma cells. *p < 0.05 vs. treatment with control-miR, ^#^*p* < 0.05 vs. treatment with control-miRZIP. **(P)** YTHDF2 gene expression in glioma clinical specimens measured by RT-qPCR. *p < 0.05 vs. normal brain tissues, ^#^*p* < 0.05 vs. WHO II glioma tissues, ^&^*p* < 0.05 vs. WHO III glioma tissues. **(Q)** Detection of PTEN gene expression in glioma clinical specimens using RT-qPCR. *p < 0.05 vs. normal brain tissues, ^#^*p* < 0.05 vs. WHO II glioma tissues, ^&^*p* < 0.05 vs. WHO III glioma tissues. **(R)** Pearson correlation analysis of YTHDF2 and PTEN expression in glioma clinical samples. **(S)** Pearson correlation analysis of YTHDF2 and miR-454-3p expression in glioma clinical samples. **(T)** The YTHDF2 protein expression after overexpression of miR-454-3p measured by Western blot analysis. All measurement data were shown as mean ± standard deviation. Data between two groups were compared by unpaired *t*-test, while comparisons among multiple groups were performed using one-way ANOVA, followed by Tukey’s *post-hoc* test. Comparison of data at different time points was analyzed using two-way ANOVA. Pearson correlation analysis was performed to observe the correlation of indicators. Experiments were repeated three times independently.

Firstly, Lenti-PTEN was constructed to inhibit PTEN, and the silencing efficiency of Lenti-PTEN was detected by western blot analysis, followed by screening of Lenti-PTEN sequence with the highest silencing efficiency (Figures 5A,B). Then Lenti-PTEN was delivered into glioma cells, and the effect of PTEN on M2 polarization of macrophages was detected in a co-culture system of glioma cells and macrophages. As depicted in Figure 5C, inhibition of PTEN in glioma cells led to significant enhancement in M2 polarization of macrophages. As reflected by RT-qPCR, immunofluorescence and ELISA, PTEN knockdown induced potent rises in the expression of MIP-3α and IL-8 in macrophages, fluorescence intensity, and their secretion in co-culture medium but reductions in the expression of IL-6 in macrophages, fluorescence intensity, and its secretion in co-culture medium (Figures 5D–G). According to western blot analysis, overexpression of miR-454-3p could promote the protein expression of PTEN, whereas inhibition of miR-454-3p could inhibit the protein expression of PTEN (Figures 5H,I). Dual-luciferase reporter assay was adopted to detect whether miR-454-3p acted on PTEN by directly binding to PTEN. However, the results suggested that neither inhibition nor overexpression of miR-454-3p significantly altered luciferase activity (Figures 5J,K). Using cyclic peptide a-amanitin (50 mM) to inhibit RNA synthesis in cells, we found that PTEN mRNA content decreased significantly in a time-dependent manner. Overexpression of miR-454-3p could inhibit the decrease of PTEN mRNA, while inhibition of miR-454-3p could enhance the decrease of PTEN mRNA (Figures 5L,M). RT-qPCR revealed that as the WHO grade of glioma increased, the expression of PTEN gradually decreased, while that of YTHDF2 gradually increased (Figures 5N–Q). Then, Pearson correlation analysis exhibited a negative correlation between YTHDF2 and PTEN expression and a negative correlation between miR-454-3p and YTHDF2 expression in the glioma tissue samples (Figures 5R,S). Additionally, Western blot analysis results displayed that overexpression of miR-454-3p markedly inhibited YTHDF2 protein expression (Figure 5T). In summary, miR-454-3p increased PTEN expression and reduced YTHDF2 expression in glioma cells.

### miR-454-3p Promotes PTEN Expression by Inhibiting m^6^A Modification of PTEN in Glioma Cells

**FIGURE 6:**
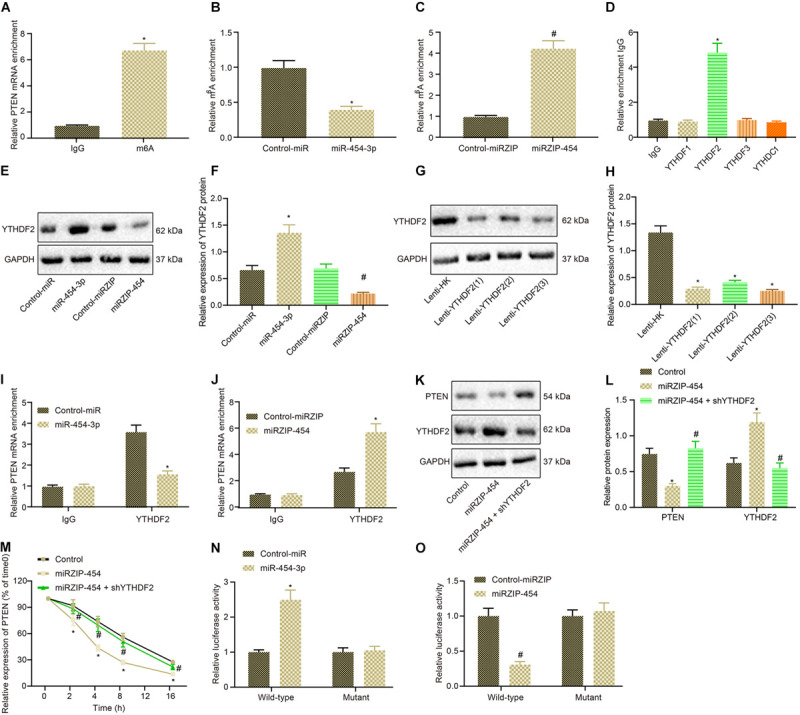
miR-454-3p depresses m^6^A modification of PTEN to upregulate PTEN in glioma cells. **(A)** Detection of m^6^A modification in glioma cells using MeRIP assay. *p < 0.05 vs. IgG antibody. **(B)** The m^6^A modification of A172 cells treated with miR-454-3p detected using MeRIP assay. *p < 0.05 vs. treatment with control-miR. **(C)** Detection of m^6^A modification in A172 cells treated with miRZIP-454 using MeRIP assay. ^#^*p* < 0.05 vs. treatment with control-miRZIP. **(D)** The binding of different m^6^A methyltransferases to miR-454-3p measured by IgG, YTHDF2, METTL14, or WTAP antibodies using RIP assay. *p < 0.05 vs. IgG antibody. **(E)** The bands of YTHDF2 protein pulled down by YTHDF2. **(F)** The binding of miR-454-3p to YTHDF2 determined by RNA pull down assay. *p < 0.05 vs. treatment with control-miR, ^#^*p* < 0.05 vs. treatment with control-miRZIP. **(G)** The efficiency of Lenti-PTEN determined using western blot analysis. **(H)** The protein expression of PTEN. *p < 0.05 vs. treatment with Lenti-HK. **(I)** The binding of PTEN to YTHDF2 in control A172 cells and A172 cells treated with miR-454-3p assessed by IgG and YTHDF2 antibodies using RIP experiment. *p < 0.05 vs. treatment with control-miR. **(J)** The binding of PTEN to YTHDF2 in control A172 cells and A172 cells treated with miRZIP-454 evaluated by IgG and YTHDF2 antibodies using RIP experiment. ^#^*p* < 0.05 vs. treatment with control-miRZIP. **(K)** Western blots of PTEN protein in A172 cells treated with miRZIP-454 and shYTHDF2. **(L)** PTEN protein expression in A172 cells treated with miRZIP-454 and shYTHDF2. *p < 0.05 vs. control A172 cells, ^#^*p* < 0.05 vs. A172 cells treated with miRZIP-454. **(M)** The mRNA expression of PTEN every 4 h after treatment of miRZIP-454 and shYTHDF2 and addition of RNA synthesis inhibitor a-amanitin (50 mM) into glioma cells, *p < 0.05 vs. control A172 cells, ^#^*p* < 0.05 vs. A172 cells treated with miRZIP-454. **(N)** The luciferase activity of the PTEN fused reporter gene in A172 cells with mutations in the YTHDF2 binding motif of the PTEN-3’ UTR after treatment with miR-454. **(O)** The activity of the PTEN fused reporter gene in A172 cells with mutations in the YTHDF2 binding motif of the PTEN-3’ UTR after treatment with miRZIP-454. *p < 0.05 vs. control-miR, ^#^*p* < 0.05 vs. control-miRZIP. All measurement data were shown as mean ± standard deviation. Data between two groups were compared by unpaired *t*-test, while comparisons among multiple groups were performed using one-way ANOVA, followed by Tukey’s *post-hoc* test. Comparison of data at different time points was analyzed using two-way ANOVA. Experiments were repeated three times independently.

We continued to verify whether miR-454-3p affected PTEN expression by affecting m^6^A modification of PTEN. Initially, m^6^A modification of PTEN was assessed in glioma cells by MeRIP assay, which identified the presence of m^6^A modification in PTEN in glioma cells, that is, the expression of PTEN was regulated by m^6^A modification (Figure 6A). Then, we examined whether miR-454-3p could affect m^6^A modification of PTEN. Treatment of miR-454-3p significantly diminished the m^6^A modification of PTEN, whereas treatment of miRZIP-454 enhanced the m^6^A modification of PTEN markedly (Figures 6B,C). Moreover, RIP experiments found that YTHDF2 was co-immunoprecipitated with miR-454-3p (Figure 6D). RNA pull-down further confirmed that miR-454-3p was co-immunoprecipitated with YTHDF2, whilst miRZIP-454 counteracted this binding effect (Figures 6E,F). Lenti-YTHDF2 sequence with the best silencing effect was selected for subsequent experiments (Figures 6G,H). RIP experiments were conducted again to confirm whether YTHDF2 could bind to PTEN. As manifested in Figures 6I,J, Compared with IgG antibody, YTHDF2 antibody could co-immunoprecipitate PTEN mRNA. The treatment of miR-454-3p into cells potently inhibited the binding of YTHDF2 to PTEN, but conversely, treatment of miRZIP-454 strikingly enhanced this binding. Western blot analysis results showed that treatment of miRZIP-454 into cells contributed to a marked decline of PTEN protein expression, while simultaneous treatment of shYTHDF2 counteracted the effect of miRZIP-454 (Figures 6K,L). Cyclic peptide a-amanitin (50 mM) RNA synthesis inhibition experiments also confirmed that shYTHDF2 neutralized the increased degradation rate of PTEN mRNA caused by miRZIP-454 (Figure 6M). The YTHDF2 binding motif (GGACH, H = A, C or U) was searched in PTEN-3′ UTR, and six sites were identified. These motifs were mutated (from 5′-GGACU-3′ to 5′-GGUCH-3′) to block the m^6^A modification pathway by which YTHDF2 recognized PTEN. Then it was found that mutation of m6A consensus sequence enhanced the activity of the reporter gene fused with PTEN and GFP in A172 cells by dual luciferase reporter assay, and treatment of miR-454-3p in WT A172 cells could achieve the same effect, whereas treatment of miRZIP-454 diminished the activity of the reporter gene fused with PTEN and GFP (Figures 6N,O). Taken together, miR-454-3p decreased m^6^A modification of PTEN and then upregulated PTEN in glioma cells.

## Discussion

It has been recently reported that EZH2 inhibition increases cell apoptosis and reduces cell proliferation in glioma (Wang et al., 2019), and it also potentially prevents macrophage-dependent disease development (Neele and de Winther, 2018). However, considering many potential miRs governed by EZH2, one challenge in understanding functional EZH2 contributions in glioma cell behaviors depends on the identification of bona fide molecular targeted miRs. In this study, we were intended to find out whether action modes of EZH2 affected glioma cells and their effects on macrophage polarization. Consequently, this study illustrated that EZH2 induced miR-454-3p DNA methylation by binding to miR-454-3p promoter to decrease miR-454-3p expression, thus upregulating YTHDF2 and m^6^A decoration of PTEN, which promoted M2 macrophage polarization in glioma.

Initially, our results found that with the increase of glioma WHO grade, EZH2 expression in glioma clinical samples showed an increasing trend. Accumulating evidences displayed the upregulation of EZH2 in glioma tissues and cells (Orzan et al., 2011; Zhang et al., 2012; Xu H. et al., 2019), which supported our results. In addition, a prior study showed that EZH2 inhibition could be involved in the therapy targeting cancer stem cells for glioma (Jin et al., 2017). Importantly, a research by Yang et al. (2017) elucidated that EZH2 upregulation caused by exogenous PVT1 resulted in promotion of cell proliferation and invasion but suppression of apoptosis in glioma. Moreover, DNA methylation is generally linked to transcriptional alterations, including silencing of tumor suppressors, to modulate glioma development (Court et al., 2019). It was observed by Bao et al. that as a histone methyltransferase, EZH2 could be recruited in the miR-454-3p promoter to induce miR-454-3p DNA methylation and thus decrease miR-454-3p expression in chondrosarcoma cells (Bao et al., 2017), which was coincident with our results that EZH2 inhibited miR-454-3p by DNA methylation. Additionally, results from a recent study unraveled that miR-454-3p downregulation was observed in glioblastoma cells and tissues and its downregulation could promote cell proliferation and repress cell apoptosis (Zuo et al., 2019). This study demonstrated that inhibition of miR-454-3p in glioma cells promoted M2 macrophage polarization, corresponding to elevated expression of II1b, Cd86, and Nos2, and reduced expression of Cd163, Ym1 and Mrc1 in macrophages. Taken together, EZH2 overexpression contributed to glioma development by inducing miR-454-3p DNA methylation and decreasing miR-454-3p expression.

It is well-known that miRs binds to target genes and post-transcriptionally inhibits target gene expression (Li et al., 2016). For instance, miR-454-3p was documented to target ATG12 to repress glioma cell proliferation and invasion (Shao et al., 2019). Besides, miR-454-3p was detected to target BTG1 to render renal carcinoma cancer cells sensitive to radiation (Wu et al., 2014). Interestingly, our data uncovered that miR-454-3p promoted PTEN expression by inhibiting m^6^A modification of PTEN through binding to YTHDF2. It is acknowledged that transcriptome m^6^A modification can be dynamic, and that the m^6^A reading protein YTHDF2 increases mRNA decay during the cell cycle (Fei et al., 2020). It was elaborated that YTHDF2 recognized the PTEN m^6^A modification pathway in gastric cancer cells (Yan et al., 2020). Findings obtained from a study reported by Chen et al. (2017) suggested the overexpression of YTHDF2 in pancreatic cancer tissues in contrast to normal tissues. Consistent with our finding, a prior study revealed that YTHDF2 expression was elevated in glioma tissues with the increase of WHO grade (Chai et al., 2019). Meanwhile, PTEN downregulation has been detected in glioma tissues by a prior research (Chai et al., 2018), which was also in line with our finding. Further investigation showed that poor PTEN expression contributed to increase of glioma cell proliferation (Liu et al., 2017). PTEN knockdown was further proved in this study to promote the polarization of M2 macrophages, which is consistent with the findings reported by Bao et al. that PTEN downregulation enhanced expression of M2 macrophage-specific markers (CD206, CD204, CD163), which thus promoted macrophage M2 polarization in glioma (Bao and Li, 2019). Therefore, EZH2/miR-454-3p/PTEN axis was involved in glioma progression, especially macrophage polarization. Our subsequent experiments documented that EZH2 upregulation contributed to M2 macrophage polarization in glioma by downregulating PTEN through miR-454-3p-dependent promotion of PTEN, characterized by IL-6 downregulation and IL-8 and MIP-3α upregulation. Glioma-associated microglia/macrophages correlate with tumor progression and anti-tumor immune responses (Roesch et al., 2018). IL-6, TNF-α, and IL-12 are markers of M1-type macrophages that can repress glioma progression (Qian et al., 2020). It is documented that M2 macrophages lead to promotion of glioma progression and immune escape through production of IL-8, and that MIP-3α increases macrophage recruitment to affect tumor growth (Xu Y. et al., 2019). Hence, EZH2/miR-454-3p/PTEN axis modulated macrophage M2 polarization in glioma.

## Conclusion

**FIGURE 7:**
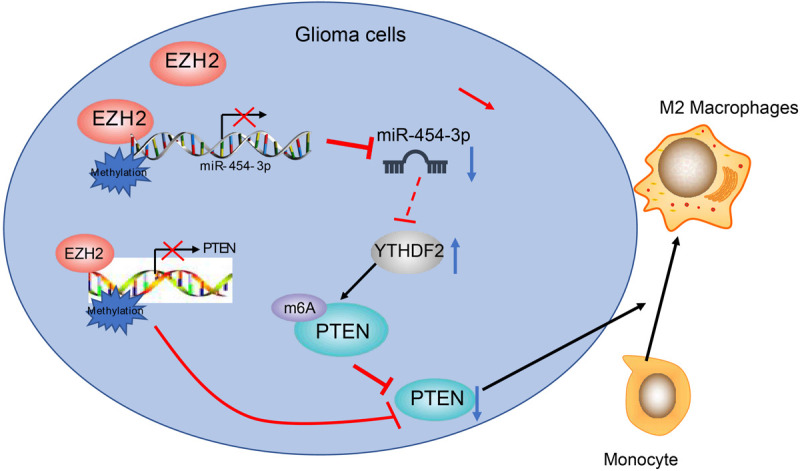
The scheme of the mechanism by which EZH2 affects glioma tumorigensis. EZH2 inhibits miR-454-3p to enhance the binding to m^6^A reading protein YTHDF2, whereby promoting m^6^A modification of PTEN and inducing M2 macrophage polarization in glioma and tumorigensis.

Conclusively, we have confirmed that EZH2 serves as an oncogene in glioma by downregulating miR-454-3p. We also demonstrate that EZH2 promotes M2 macrophage polarization in glioma by increasing m^6^A modification of PTEN via inhibiting miR-454-3p (Figure 7). We speculate that EZH2 and miR-454-3p may be promising new directions in the development of therapeutic treatments for glioma.

## Data Availability Statement

The original contributions presented in the study are included in the article/Supplementary Material, further inquiries can be directed to the corresponding author.

## Ethics Statement

The studies involving human participants were reviewed and approved by the First Hospital of Jilin University. The patients/participants provided their written informed consent to participate in this study. The animal study was reviewed and approved by the First Hospital of Jilin University.

## Author Contributions

BQ and CY designed the study and contributed to drafting the manuscript. ZZ and HC collated the data, carried out data analyses, and produced the initial draft of the manuscript. All authors have read and approved the final submitted manuscript.

## Conflict of Interest

The authors declare that the research was conducted in the absence of any commercial or financial relationships that could be construed as a potential conflict of interest.

## Footnotes

1 http://genome.ucsc.edu/index.html

2 http://www.urogene.org/index.html

## Abbreviations

miR-454-3p: microRNA-454-3p
EZH2: Enhancer of zeste homolog 2
YTH: YT521-B homology
PTEN: Phosphatase and tensin homolog
RT-qPCR: Reverse transcription quantitative polymerase chain reaction
GFP: green fluorescent protein
PBS: phosphate buffer saline
ELISA: Enzyme-linked immunosorbent assay
IL-8: interleukin-8
MIP-3 α: macrophage inflammatory protein-3 α
BCA: bicinchonininc acid
3′-UTR: 3′-untranslated region
WT: wild type
MUT: mutant type
β-gal: β-galactosidase
HE: Hematoxylin-eosin
IgG: Immunoglobulin G
DAPI: diamidino-2-phenylindole
RIPA: radio-immunoprecipitation assay
ECL: electrogenerated chemiluminescence
SPF: specific pathogen-free
RIP: RNA immunoprecipitation
MeRIP: methylated RNA immunoprecipitation.
