# Supplementary figures and images for: EZH2-Inhibited MicroRNA-454-3p Promotes M2 Macrophage Polarization in Glioma
Source: Front Cell Dev Biol. 2020 Dec 9;8:574940. doi: 10.3389/fcell.2020.574940 (PMC7755639; doi:10.3389/fcell.2020.574940)

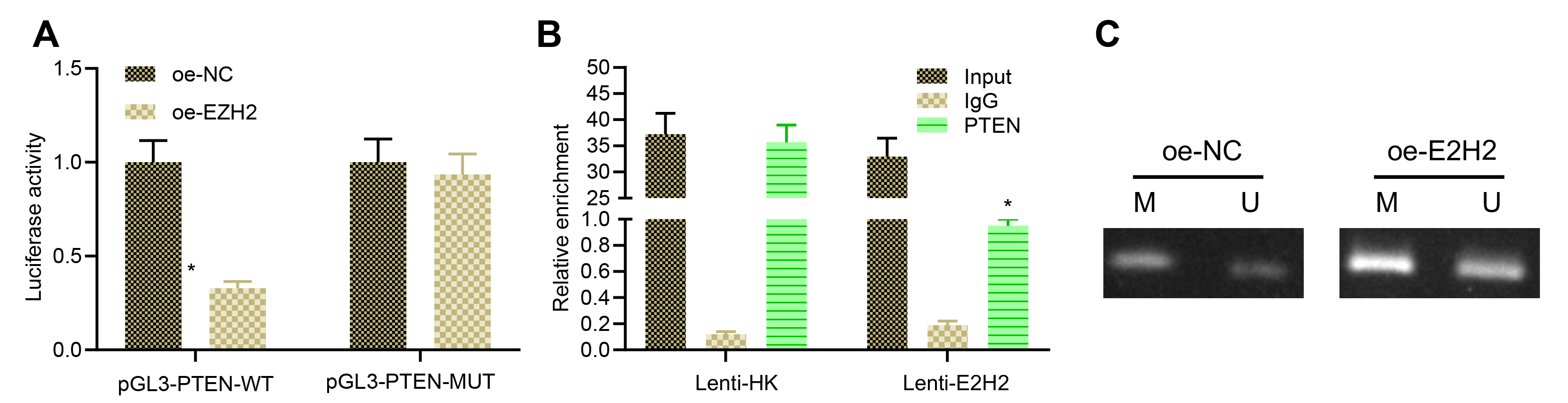

Supplement: Supplementary file 1 [file Image_1.JPEG]

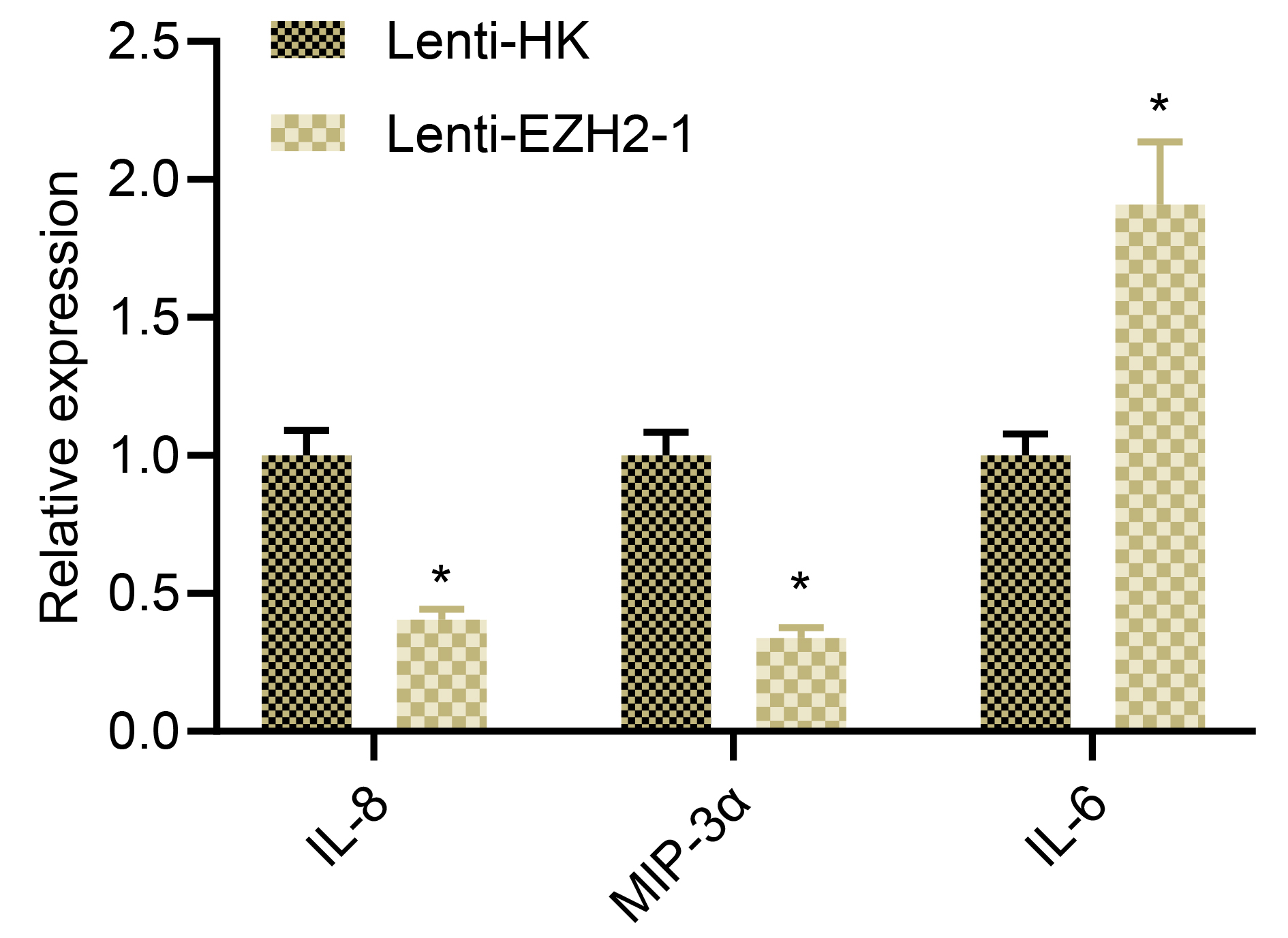

Supplement: Supplementary file 2 [file Image_2.JPEG]
