# Supplementary material for: EZH2-Inhibited MicroRNA-454-3p Promotes M2 Macrophage Polarization in Glioma
Source: Front Cell Dev Biol. 2020 Dec 9;8:574940. doi: 10.3389/fcell.2020.574940 (PMC7755639; doi:10.3389/fcell.2020.574940)
Supplement: Supplementary file 3 [file Table_1.DOCX]

**EZH2 directly binds to the PTEN promoter to suppress the expression of PTEN**

The results of dual luciferase reporter assay showed that overexpression of EZH2 reduced the activity of the PTEN promoter in glioma cells (Fig. S1A). ChIP results revealed that EZH2 could directly bind to the PTEN promoter region (Fig. S1B). Furthermore, MS-PCR results exhibited that the overexpression of EZH2 promoted the methylation status of PTEN in glioma cells (Fig. S1C). The above results indicate that EZH2 directly binds to the PTEN promoter thus promoting PTEN methylation to suppress the expression of PTEN. As revealed by RT-qPCR, silencing of EZH2 using Lenti-EZH2-1 significantly reduced the expression of MIP-3α and IL8, while increased the expression of IL6 (Fig. S2).

**LEGENDS**

**Fig. S1** EZH2 promotes the methylation of PTEN. A, The binding relationship between EZH2 and the PTEN promoter determined by a dual luciferase reporter assay. * *p* < 0.05 *vs.* treatment with oe-NC. B, The binding relationship between EZH2 and the PTEN promoter identified by ChIP. * *p* < 0.05 *vs.* treatment with Leti-HK. C: The methylation status of PTEN determined by MS-PCR. All measurement data were shown as mean ± standard deviation. Comparisons among multiple groups were performed using one-way ANOVA, followed by Tukey's post hoc test. Comparison of data at different time points was analyzed using two-way ANOVA. Experiments were repeated three times independently.

**Fig. S2** EZH2 silencing by Lenti-EZH2-1 reduces the expression of MIP-3α and IL8 and increases that of IL6. The expression of IL8, MIP-3α, and IL6 in cells treated with Lenti-HK or Lenti-EZH2-1 determined by RT-qPCR. Experiments were repeated three times independently.
